# Book Reviews

**Published:** 1906-06

**Authors:** 


A TREATISE ON DISEASES OF THE SKIN. For the use of advanced Students and Practitioners. By Henry W. Stelwagon, M.D., Ph.D., Professor of Dermatology, Jefferson Medical College, Philadelphia; and Clinical Professor of Dermatology, Woman's Medical College, Philadelphia. Fourth edition, revised. Handsome octavo of 1135 pages, with 258 text-illustrations, and 32 full-page lithographic and half-tone plates. Philadelphia and London: W. B. Saunders & Company, 1905. Cloth, $6.00 net; sheep or half morocco, $7.00 net.

Four large editions of Dr. Stelwagon's work have been required in three years. Surely such a sale bespeaks a book of unusual merit. Notwithstanding the frequency of editions, Dr. Stelwagon has not lost his opportunity to bring his book up to the latest knowledge. The therapeutic use of the Rontgen rays, high-frequency current, and Finsen light have been accorded the increased attention their young growing importance deserves. We notice the addition of new text-cuts, some thirty-eight in number, and six additional insert plates, all up to the high standard set by the text. The author, by the judicious elimination of redundant material, has kept the size of his book much as before, the increase being only some twenty pages. Indeed, it is remarkable the epigrammatic way that Dr. Stelwagon has of saying things—a style most desirable both in a text-book and a reference work for the busy practitioner.

---

THE SIGN OF INTERNAL DISEASE. By Pearce Kintzing, B.S.C., M.D., Professor of Physical Diagnosis and Diseases of the Heart in the Maryland Medical College, Baltimore, Md. Published by the Cleveland Press. Price —.

The work is a compilation in book form of the author's lectures, giving in a thoroughly practical manner the methods best adapted to the needs of the student.

The work embraces, in addition to the methods of examination as applied to the chest, the differentiation between the various abdominal troubles.

The chapters on examination of blood, urine and faeces are complete and up to date.

The work should prove useful to the practitioner, as well as the student.

---

MESSRS. D. APPLETON & Co. announce a new revised edition of the popular work on Accident and Injury, and Their Relations to Disease of the Nervous System, by Pearce Bailey, A.M., M.D., of New York.

The first edition of this work was printed in 1898. The present revision required a recasting of the entire book, therefore the present volume is printed from new plates. It is a thorough revision in every sense of the word, in fact, a new book, and the title giving this revision, namely, "Disease of the Nervous System Resulting from Accident and Injury," we believe to be a more correct one than the one used when the book was first published. A number of illustrations have been added, and many old ones have been displaced by new.

Dr. Bailey is an authority upon the subject covered in his book, especially upon the medico-legal questions.

The author says in his preface: "Those subjects most fully described in text-books on surgery are dismissed with briefest mention. The late effects of brain injuries, for example, receive more notice than the acute symptoms." For this reason the book is of the greatest interest, not only to the surgeon and neurologist, but to the general practitioner.

---

IT is surprising how much information can be derived by *abdominal palpation conducted with the patient in a hot bath,* the temperature of the water being gradually raised to 105° F. It usually secures as much relaxation as does the administration of an anesthetic, sometimes even more. In addition to the avoidance of the dangers and the disagreeable features of narcosis, it has the important advantage that the patient is able to call the examiner's attention to sensitive areas. — *American Journal of Surgery.*

